# Customized Root-Analogue Implants: A Review on Outcomes from Clinical Trials and Case Reports

**DOI:** 10.3390/ma14092296

**Published:** 2021-04-29

**Authors:** Telma Dantas, Sara Madeira, Michael Gasik, Paula Vaz, Filipe Silva

**Affiliations:** 1 CMEMS (Center for Micro Electro Mechanical Systems), University of Minho, 4800-058 Guimarães, Portugal; saracsoaresm@hotmail.com (S.M.); fsamuel@dem.uminho.pt (F.S.); 2 MIT Portugal Program—School of Engineering, University of Minho, 4800-058 Guimarães, Portugal; 3 School of Chemical Engineering, Aalto University Foundation, 02150 Espoo, Finland; michael.gasik@aalto.fi; 4 Fixed Prosthodontics, Genetics—Faculty of Dental Medicine, University of Porto, 4200-135 Porto, Portugal; cs.paula.vaz@gmail.com

**Keywords:** root-analogue implants, custom-made, CAD/CAM technology, zirconia, titanium, clinical trial

## Abstract

(1) It is estimated that 10% of the world’s population will need a dental implant in their lifetime. Despite all the advances in the comprehension of dental implant designs, materials and techniques, traditional implants still have many limitations. Customized root-analogue implants are, therefore, gaining increased interest in dental rehabilitation and are expected to not only preserve more hard and soft tissues but also avoid a second surgery and improve patient overall satisfaction. In this sense, the aim of this review was to collect and analyse the clinical trials and case reports on customized root-analogue implants available in the literature; (2) This review was carried out according to the PRISMA Statement. An electronic database search was performed using five databases: PubMed, Google Scholar, Medline, Science Direct, and Scopus. The following keywords were used for gathering data: custom-made, dental implants, root-analogue, anatomical, customized and tooth-like; (3) 15 articles meeting the inclusion criteria—articles reporting clinical trials, case reports or animal studies and articles with root-analogue implants and articles with totally customized implant geometries—were selected for the qualitative synthesis. The design and manufacturing techniques, implant material and surface treatments were assessed and discussed; (4) The performance of some root-analogue implants with specific features (i.e., macro-retentions) was successful, with no signs of infection, periodontitis nor bleeding during the follow-up periods.

## 1. Introduction

Dental implants are an attractive option for replacing missing teeth, providing many advantages, reliability and comfort for improving quality of life [1]. There is a variety of different implants systems on the market [2,3] and some companies are already offering an implant selection system for their customers. However, the approaches aiming at implementation of completely customized dental implants are still uncommon [4]. Osseointegration has been defined as a direct and functional connection between bone and an artificial implant [5]. Traditional implants have a cylindrical or tapered geometry with threads along the screw length and over the placed abutment, followed by the crown (for a single tooth). Due to the geometry and design, they may only provide limited options for available implant length, diameter, and thread parameters, and, therefore, cannot completely meet the personalized requirements of every patient [4,6]. This lack of proper congruency between the implant and the socket bone can eventually lead to implant failure due to stability loss and osseointegration [7,8]. To overcome this problem, novel approaches are being evaluated to manufacture customized root implants which are explicitly tailored to each patient’s condition. This is expected to reduce the bone and soft-tissue trauma and promote a better primary stability, being thus a promising alternative for dental rehabilitation [6,7]. Additionally, the placement of such root-analogue implants (RAI) is a minimally invasive procedure, since they do not usually require bone drilling, sinus lifting, bone augmentation or other traumatic procedures [9]. Root-analogue implants were first described by Hodosh et al. back in 1969 [10]. A polymethacrylate implant was developed and clinically tested at that time, but outcomes were not satisfactory. Some years later, Lundgren D. et al. [11] reintroduced the topic by developing a titanium RAI and testing it in beagle dogs. Results revealed that the use of titanium instead of a polymeric material led to a success rate of 88% (28 of the 32 implants were successfully osseointegrated).

Titanium remains still as “the gold standard” metallic material for dental implants. However, an increased concern with aesthetical issues has led to an increased interest in ceramic materials, namely zirconia, for such applications [12]. Apart from its tooth-like colour, its high corrosion resistance, biocompatibility and high wear resistance make zirconia a promising material for dental implants [13]. This is being already deployed in dental practice with zirconia abutments [14,15]. Despite the recent developments in the design and implementation of totally customized root-analogue implants, reliable data on the long-term use of RAI in humans are still scarce.

The present review aims on collection and analysis of the few clinical trials and case reports available in the literature on customized root-analogue implants. The objective is to compare their clinical performance together with additional parameters of these implants (materials used, surface treatments, design and manufacturing techniques). Also, aspects related to the surgical procedure, such as extraction of the teeth, the time between tooth extraction and implant placement, the time between placement and final reconstruction, and main biological outcomes were accessed.

## 2. Methods

### 2.1. Search Strategy

This review was carried out according to the PRISMA Statement (Preferred Reporting Items for Systematic Reviews and Meta-Analyses) [16]. An electronic database search was performed using five databases: PubMed, Google Scholar, Medline, Science Direct, and Scopus. To conduct the search, Boolean operators such as “AND” and “OR” were used to correlate the keywords. The following keywords were explored and inserted with the field tag (Title/Abstract/Keywords): “custom-made”, “dental implants”, “root-analogue”, “anatomical”, “customized” and “tooth-like”. In addition, the references of review articles, as well as articles published in the International Journal of Oral and Maxillofacial Implants, International Journal of Oral and Maxillofacial Surgery, International Journal of Prosthodontics, Journal of Periodontology, Journal of Oral Implantology, Journal of prosthetic dentistry, and International journal of prosthodontics, were manually searched to include all the relevant articles available in the literature.

### 2.2. Study Selection

A screening process was conducted over the titles and abstracts retrieved by the databases search, to select the articles for full-text reading. From this screening process and after duplicates removal, 64 articles were selected for full-text reading. In order to assess their eligibility to be included in this review, the following inclusion criteria were applied: (1) articles written in English, (2) articles dated from 1990 to 2020, (3) articles reporting clinical trials, case reports or animal studies, (4) articles with root-analogue implants, (5) articles with totally customized implant geometries. Articles not meeting these inclusion criteria were excluded from the review (articles comprising just a review were also excluded). Articles that met the inclusion criteria were entirely read and analysed considering the aim of this review.

### 2.3. Data Collection and Extraction

The information extracted from each article was divided into two main groups: the implant characterization and the aspects related to the clinical trial/case report. As far as the implant is concerned, a brief summary of the materials, design and manufacturing techniques, as well as surface modifications, were analysed. For the second, aspects related to the clinical procedure were also evaluated (the number of subjects, the number of implants per subject, the treated tooth, the time between tooth extraction and implant placement, the time between the implant placement and final reconstruction, the follow-up periods, and the main technical or biological complications).

## 3. Results

### 3.1. Study Selection

**Figure 1:**
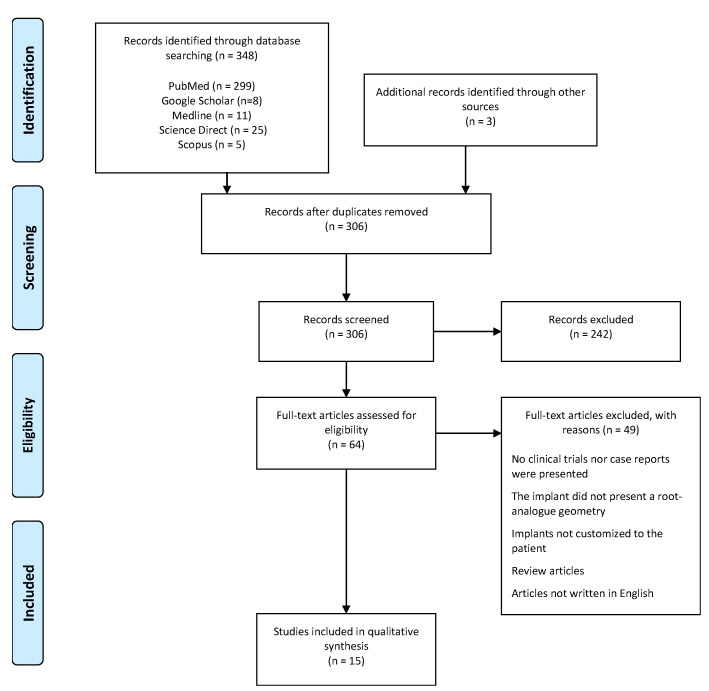
Search strategy flowchart, adapted from [16].

Automatic and manual database searches resulted in a total of 348 records. After duplicate removal, 306 titles and abstracts were evaluated and a total of 64 full texts were selected to assess their eligibility to be included in this review. Of these 64 publications, 49 could not be included in the final analysis. These articles were excluded for one or more of the following reasons: (1) they did not present any clinical trial nor case data in the report; (2) the implant did not present an RAI geometry; (3) the implant was not actually customized to the specific patient; (4) they were purely review articles. The application of the exclusion criteria resulted, therefore, in 15 articles to be evaluated and analysed in detail for this work. In Figure 1 the PRISMA flowchart used in this selection process is shown. Table 1 lists these selected articles’ titles, authors and year of publication. As can be seen, articles found in the literature and included in this review are dated from 1992 to 2018.

After a careful analysis of these fifteen articles, the authors were able to extract a lot of information to be analysed and compared. Below two main groups are shown in more detail: the data of the RAI itself (used materials, design and manufacturing techniques, surface treatments) and the parameters of the clinical trials or report cases (number of patients, number of implants, treated teeth, clinical procedures, follow-up periods, etc.).

### 3.2. Design and Manufacturing Techniques, Implant Material and Surface Treatments

The dental implants developed in the scope of these investigations present different materials and design/manufacturing techniques. Also, different surface treatments, to promote stability and osseointegration are reported. In Table 2 it is possible to observe a summary of the root-analogue implants’ characteristics.

Almost half of the implants used in these works (7 out of 15) are totally made of titanium or its alloys, namely commercial purity (CP) titanium [11], grade II titanium [17,18] and Ti6Al4V [23,24,25,27]. On the other hand, six of the analysed articles aimed to develop dental implants 100% made of zirconia [19,20,21,22,26,28]. Additionally, two of the publications found in the literature reported dental implants consisting of both titanium and zirconia [29,30]. These two RAI have a titanium root with a zirconia abutment on the top.

For the design of RAI two main differences were detected in techniques. Part of the studies described RAI design after the tooth removal, for example, by laser scanning of the original root [11,17,18,19,20,21,22,26,28]. In that approach [19,20,21,22,26,28] macro-retentions were designed on the implant surface, to ensure the implants’ stability. Additionally, a crown stump was designed for a posterior connection to the final restoration. In the second technique, RAI was designed before tooth extraction [23,24,25,27,29,30], aiming to reduce the surgical interventions. This strategy comprises the acquisition of the 3D CT (Computed Tomography) images of the patient with posterior image treatment with the help of 3D software.

Milling of the pre-designed models presents the most common manufacturing method [11,17,18,19,20,21,22,26,28,29,30]. Four clinical studies reported manufacturing with Direct Laser Metal Sintering (DLMS), also referred to as Direct Laser Metal Forming (DLMF) [23,24,25,27]. For the implants consisting of two different materials (such as titanium fixture and zirconia abutment), a biocompatible glass solder was used to create a permanent bond between the implant root and abutment [29,30].

**Figure 2:**
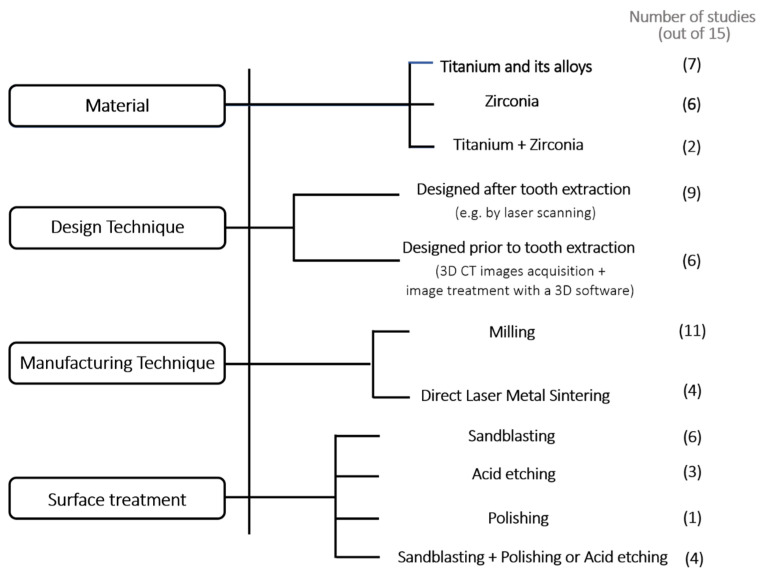
Schematic representation of the materials, design and manufacturing techniques, and surface treatments of the selected and analysed studies.

Surface treatment techniques usually involve sandblasting [17,18,19,20,21,22,26,28,29,30] but some also report other complementary surface treatments to sandblasting, namely polishing and acid etching [17,18,29,30]. Three studies included exclusively acid etching on their implant surface [23,24,25], which correspond to the Ti6Al4V implants manufactured by DLMS, and one simply polished the implant surface [11]. In Figure 2 It Is possible to observe a schematic representation that summarizes the aforementioned characteristics of the selected studies, for a better understanding.

### 3.3. Clinical Trials/Case Reports Summary

In this section of the work, the authors summarized the main parameters and procedures of the clinical trials/case reports (Table 3). Among the selected articles, two-thirds (10 out of 15) correspond to a case report study, meaning that only one patient and one tooth were evaluated [18,19,21,22,23,24,26,27,28,29]. As far as the clinical trials are concerned [11,17,20,25,30], two of the articles correspond to pre-clinical trials [11,17], which means that the studies were performed in animals, namely in beagle dogs (4 subjects, with eight implants each) and monkeys (three subjects, with four implants each). The other clinical trials were performed in eighteen [20], fifteen [25], and five subjects [30]. The treated tooth varied from article to article.

After a careful analysis of the clinical procedures carried out in these studies, it was possible to conclude that almost half of the strategies (in 7 articles) perform the implant placement immediately after tooth extraction, in the same surgical procedure [17,23,24,25,27,29,30]. In the pre-clinical trial performed in beagle dogs [11], four teeth were extracted on the same day of the implant placement, whereas the other twenty-eight teeth had been extracted two weeks before. In the remaining articles, implants were placed between 1–8 days after the tooth extraction. All authors mention that the implants were placed into the socket under finger pressure and subsequent gentle tapping with a hammer and a mallet. Additionally, primary stability was commonly checked by palpation and percussion. As far as the final restoration is concerned, three to four months is the most reported time to wait between the placement of the implant and the crown, being reported in ten articles [19,20,21,22,23,24,25,27,28,30]. The follow-up periods reported in these studies range from six months to three years, being one year the most common period of time [23,24,25,27,30]. In the results section of the analysed articles, it is possible to find which were the main clinical and/or biological complications that occurred during the follow-up period, as well as the overall performance of the implant and whether success was achieved or not.

After evaluating the performance of the implants of the case reports [18,19,21,22,23,24,26,27,28,29], it is possible to observe that outcomes of the different studies are however rather similar (Table 3). In fact, none of the implants was lost after the follow-up period. All the authors, except Heydecke et al. reported good implant stability, no bleeding, no signs of periodontitis nor bone recession. In study [18], bony resorption and soft tissue recession led to a slight discoloration of the marginal peri-implant mucosa. Excellent aesthetic results were reported in the other case reports, mainly in the ceramic-based implants. In general, all authors mentioned a quite satisfactory implant performance during the follow-up period of the case reports. This is an interesting observation especially for the peri-implantitis appearance. It is well-known that the acid etching or electrochemical treatment of titanium aimed to generate an anatase layer is very beneficial for the prevention of biofilm formation [33,34]. However, this is not straight possible for zirconia and there have been concerns that full zirconia implants might be not so resistant to biofilm formation. This issue was recently demonstrated to be possible to solve with a thin coating of zirconia with TiO_2_-forming formulations [35], but no such treatment was used in the reported cases. It looks that this matter might need an additional investigation.

For the less satisfactory clinical trials the outcomes were mainly limited due to the higher number of tested implants (Table 3). In the pre-clinical trial performed in beagle dogs [11], 2 of the 32 implants were lost in the first week. Other two implants were not clinically stable after one year. The remaining 28 implants fulfilled the clinical criteria for osseointegration. In the other pre-clinical trial [17], performed in monkeys, some complications were also observed. Despite 4 of the 12 implants being exposed after a certain period of time, none of the implants was lost. However, the follow-up period of this study was too short (6 months), which may be influencing these results. In the clinical trial performed by [20] two different implants were evaluated: one designed by laser scanning of the original tooth root (Group A) and other designed also by laser scanning and with the incorporation of macro-retentions in its surface (Group B). The results of the two tested groups are very different. In Group A, five (out of six) implants were lost. The incorporation of the macro-retentions (Group B) led to more satisfactory results since only one implant was lost (out of twelve). The other eleven implants presented no signs of infection nor mobility. F. Mangano et al. [25] carried out a clinical trial in fifteen patients. After a one-year follow-up, none of the implants was lost and all of them were stable with no signs of infection. In the last analysed clinical trial [30] one (out of five) implant was lost after four weeks, due to implant mobility. The other four implants were considered successful after a one-year follow-up.

## 4. Discussion

This review has evaluated the clinical performance of customized root-analogue implants (RAI). The most differentiating aspects found in the selected literature were the type of clinical study (case reports, clinical trials or pre-clinical trials), the implant material(s) and the designing techniques.

Two materials-titanium alloys) and zirconia-were the only materials found in RAI used in these studies. The excellent mechanical properties of titanium, its biocompatibility, high corrosion resistance and low weight, are well known for this material as a solution for dental implants [36]. However, its colour together with the possible long-term corrosion and release of ions to the body environment has led to an increase in the interest in zirconia as an alternative to this metallic material [37].

Zirconia is characterized by its biocompatibility, sufficiently low bacterial affinity (yet higher than treated titanium), high mechanical flexural and compressive strengths, excellent wear resistance, and adjustable white colour, being a promising solution to overcome the aesthetic issues caused by metallic dental implants [38]. The implants’ surface finishing, namely their roughness has been proved to have a huge influence on the implant osseointegration. Some published studies indicate that rough surfaces promote a faster osseointegration comparing to smooth ones [39,40,41], and many techniques have been applied for the creation of the desired implant roughness. The most common found in literature, and also in the clinical studies of this review are sandblasting and acid etching [42,43]. Sandblasting followed by an acid etching treatment might be considered as the gold standard surface modification in the dental implants market world [44]. On the other hand, it has been shown [34,45,46] that using simple roughness value as a single parameter is a significant oversimplification, as other factors together with porosity, hydrophilicity, nano- and macrotopology are important for implant success (at least, for metallic titanium materials).

One of the biggest differences found in the evaluated literature was the implant designing technique. Some authors opted to scan the original tooth by laser, others designed the implant before tooth extraction, using the patient radiographic (CT) images, followed by 3D image manipulation, and posterior implant milling. This approach, known as computer-aided design/computer-aided manufacturing (CAD/CAM) technology, has become increasingly popular in the dentistry field over the past years [47]. In fact, many dental offices worldwide have been trying to implement modern IT solutions in their daily practice in order to reduce costs, work more efficiently, and increase patient satisfaction [48]. Modern CAD/CAM solutions seem to be the future of the dentistry field, namely for the customization of dental implants.

The success of an implant is known to be directly dependent on its osseointegration process. This process requires an initial interlocking between the alveolar bone and the implant (primary stability) and later, biological fixation through continuous bone remodelling toward the implant (secondary stability) [49]. There are key factors that influence implant stability: surface roughness (as previously mentioned), the congruity between the implant and bone, the period of time between tooth extraction and implant placement, bacterial adhesion, among others [50]. The majority of the studies that are being analysed reported ideal primary stability, where a perfect correspondence between the implant and the post-extraction socket was observed. However, in one study [17] some implants could not be inserted to the intended depth leading to implant exposures. Nevertheless, none of those implants was lost.

For the latency time (between tooth extraction and implant placement), a healing period of 6–9 months was previously recommended (a late implant placement). Later, insertion of implants after 2–3 months was suggested (a delayed implant placement), and more recently, immediate implantation has been clinically tested too [7]. Despite new interest in immediate implant insertion, the literature reports it encompassing two main problems: (a) maintaining the implant primary stability and (b) preventing soft tissue ingrowth during the healing period [7]. Additionally, it is might to higher infection risks, flap dehiscence over the extraction site, and incongruity between the socket wall and the implant [3,14,33]. The articles evaluated in the scope of this review performed the implant placement immediately after tooth extraction or a few days later. However, and contrarily to what was reported in the literature, it seems that this strategy did not trigger any specific side effects nor biological complications. In fact, primary stability was achieved in most of the tested implants.

Another aspect that is worth discussing is the incorporation of macro-retentions on the implants’ surface. Some authors mention the incorporation of these protrusions on the implant surface aiming to promote an improved attachment to the bone and consequently improved mechanical stability. Pirker et al. [20] have even compared the performance of RAI with and without macro-retentions, and results were clearly conclusive: RAI without the macro-retentions (*n* = 5) were suddenly lost, without prior pain or infection, in the first 128 days. On the other hand, among the RAI with macro-retentions (*n* = 12), only one was lost. These results were later corroborated in one study performed by Moin D. et al. [51] that analysed, by means of FEA (Finite Element Analysis), the influence of 5 custom root-analogue implant designs on the stress distribution of peri-implant bone.

The results of this study revealed that the addition of macro-retentions to an RAI standard design would have a positive effect on the stress distribution, reduce the concentration of bone stress, and provide a better primary stability. Despite being a promising alternative, the authors believe that the macro-retentions alone may not be enough to ensure implant mechanical stability. Since the RAI geometry is characterized by its conical shape, there is a risk that the implants may tend to be expelled and hence other strategies to avoid this risk should be further explored and developed. It is also notable, that FEA analysis conventionally used in dentistry usually suffers from an oversimplification of tissue properties, which are not well known (especially for soft tissues), and where anisotropy is seldom considered [52,53]. The advantage of the linear elastic models is of course in the provision of simple and direct prediction of the tissues properties for the sake of the computational efficiency but the usefulness of such data is very questionable (e.g., “elastic modulus of mucosa” ranging from 0.1 to 680 MPa [54]). FEA outcomes should be considered as a complement to the clinical studies, aiming to better understand the influence of some variables on the implants’ clinical performance.

Together with the aforementioned mechanical features, there are also some mechanobiological aspects expected to improve the implants’ osseointegration. Some reported techniques are being developed and evaluated aiming to promote the infiltration and supply of nutrients and fluids around dental implants, consequently inducing vascularization at the implant’s surface [55]. However, in this review not much information regarding these biological stimuli has been found: only one article referred that small perforations were created in the palatal tissue of the socket to stimulate bleeding [29]. Other techniques, such as the creation of hydrophilic surfaces or the incorporation of micro-channels on the implant’s surface would also have a positive impact on the implant’s vascularization [56], and eventually needed to be further explored to reveal their potential clinical benefits.

Despite the satisfactory clinical results observed in the selected articles, none of these solutions is widely available on the market—the manufacturing companies and dentists tend to prefer standard products with lower associated costs; most of the dental clinics do not have the necessary equipment (CBCT) for the design of such customized solution; despite its weaknesses, conventional dental implants have been reported with success rates of 90–95% for 10 years follow up periods [57] based on current definitions of success, which are questionable in the opinion of the authors of this paper.. Authors believe that these factors may be hindering the worldwide practice of such dental treatment and studies should proceed, focusing on the implementation of RAI in the global dental market.

The main findings of this review show that:

- Titanium and zirconia are the selected materials for the manufacturing of RAI.
- CAD/CAM technology followed by surface treatments such as sandblasting has been the preferred manufacturing technique for such applications.
- The clinical outcomes of the analysed studies suggest that further investigations should be performed aiming to evaluate whether RAI may be considered a promising solution for the replacement of missing teeth or not.

The limitations of this study rely, mainly, on the reduced number of articles meeting the inclusion criteria.

## 5. Conclusions

This review includes 15 identified clinical studies and cases with RAI. These studies have a high heterogeneity of follow-up periods, sites, techniques and thus it is difficult to compare results and draw definitive conclusions about validated RAI outcomes. Nevertheless, some general considerations and trends in RAI application can be made:

- Clinically tested RAI are made of titanium and/or zirconia; no other materials were reported for the analysed period.
- CAD/CAM is an effective technology to design and manufacture RAI and it is being implemented in the dental practice;
- Sandblasting and/or acid etching on the implant surface seems to be effective in promoting the implant osseointegration;
- The addition of macro-retentions on the implant surface induces a positive effect on the stress distribution in the bone surrounding the implant. However, this strategy alone may not be enough to promote the implant mechanical stability, due to its conical geometry;
- Immediate implant placement may be considered successful in RAI, unless there are no clinical contraindications;
- The performance of some RAI with specific features on its surface, namely the incorporation of macro-retentions, was proved to be successful, with no signs of infection, periodontitis nor bleeding during the follow-up periods.

Given the results of the evaluated clinical studies, customized root-analogue implants (cRAI) may be the future of dental rehabilitation. However, and as expected, the literature on the clinical performance of these implants is still scarce, and further well-designed clinical studies, namely long-term randomized controlled trials, are required to corroborate these findings.

## Figures and Tables

**Table 1:** Articles included in the review.

Author/Year	Title
(Lundgren, et al., 1992) [11]	Healing-in of root analogue titanium implants placed in extraction sockets. An experimental study in the beagle dog
(Kohal et al., 1997) [17]	Custom-made root analogue titanium implants placed into extraction sockets. An experimental study in monkeys
(Heydecke et al., 1999) [18]	Optimal aesthetics in single-tooth replacement with the Re-Implant system: a case report
(Pirker and Kocher 2008) [19]	Immediate, non-submerged, root-analogue zirconia implant in single tooth replacement
(Pirker and Kocher 2009a) [20]	Immediate, non-submerged, root-analogue zirconia implants placed into single-rooted extraction sockets: 2-year follow-up of a clinical study
(Pirker and Kocher 2009b) [21]	True anatomic immediate dental implant method a clinical case
(Pirker et al., 2011) [22]	Immediate, single stage, truly anatomic zirconia implant in lower molar replacement: A case report with 2.5 years follow-up
(Mangano et al., 2012) [23]	Custom-made, root-analogue direct laser metal forming implant: a case report
(Figliuzzi and Mangano 2012) [24]	A novel root analogue dental implant using CT scan and CAD/CAM: selective laser melting technology
(Mangano et al., 2014) [25]	Immediate, non-submerged, root-analogue direct laser metal sintering (DLMS) implants: a 1-year prospective study on 15 patients
(Pirker and Kocher 2015) [26]	Root analogue zirconia implants: true anatomical design for molar replacement—a case report
(Figliuzzi et al., 2016) [27]	A Direct Metal Laser Sintering (DMLS) root analogue implant placed in the anterior maxilla: case report
(Patankar et al., 2016) [28]	Immediate, non-submerged root analogue zirconia implant in single rooted tooth replacement: case report with 2 years follow-up
(Pour et al., 2017) [29]	Innovative single-tooth replacement with an individual root-analogue hybrid implant in the aesthetic zone: case report
(Moin et al., 2018) [30]	Immediate non-submerged custom root analogue implants: a prospective pilot clinical study

**Table 2:** Summary of the selected articles: implant material, design and manufacturing techniques and implant surface treatments.

Author, Year [Reference]	Material	Design Technique	Manufacturing Technique	Surface Treatment
Lundgren, et al., 1992 [11]	Commercial purity (CP) titanium	Copying the original tooth, with the help of a detection needle [31]	Milling	Polishing
Kohal et al., 1997 [17]	Grade II titanium	Laser scanning of the original tooth	Milling	Sandblasting with Al_2_O_3_ (500 µm)—intraosseous Polishing-extraosseous
Heydecke et al., 1999 [18]	Grade II titanium	A root model was created using a silicon putty material, based on the extraction socket. Its geometry was modified to perfectly fit in the alveolus and posteriorly laser scanned. The surface of the root analogue implant has a honey-comb pattern [32,33]	Milling	Sandblasting with Al_2_O_3_ (500 µm)—intraosseous Polishing-extraosseous
Pirker and Kocher 2008 [19]	Zirconia	Laser scanning of the original tooth root. Macro-retentions were designed on the implant surface. A crown stump was designed for later connection to the crown	Milling	Sandblasting
Pirker and Kocher 2009a [20]		Laser scanning of the original tooth root (Group A) and laser scanning of the original tooth root plus macro-retentions on the surface (Group B)		
Pirker and Kocher 2009b [21]		Laser scanning of the original tooth root. Macro-retentions were designed on the implant surface. A crown stump was designed for later connection to the crown		
Pirker et al., 2011 [22]				
Mangano et al., 2012 [23]	Ti6Al4V	DICOM datasets sent to a 3D reconstruction software, following by segmentation and a 3D reconstruction of the non-restorable root. A “virtual extraction” was performed, isolating the root as an STL file sent to a reverse-engineering software where the root was processed, and the prosthetic abutment was added. The diameter of the implant neck was reduced in the area in contact with the thin buccal bone	Direct Laser Metal Forming (DLMF)	Acid etching (50% oxalic acid and 50% maleic acid)
Figliuzzi and Mangano 2012 [24]				
Mangano et al., 2014 [25]				
Pirker and Kocher 2015 [26]	Zirconia	Laser scanning of the original tooth root. Macro-retentions were designed on the implant surface. A crown stump was designed for later connection to the crown	Milling	Sandblasting
Figliuzzi et al., 2016 [27]	Ti6Al4V	DICOM datasets were sent to a 3D reconstruction software, following by segmentation and a 3D reconstruction of the non-restorable root. A “virtual extraction” was performed, isolating the root as an STL file sent to a reverse-engineering software where the root was processed, and the prosthetic abutment was added. The diameter of the implant neck was reduced in the area in contact with the thin buccal bone	Direct Metal Laser Sintering (DMLS)	No information
Patankar et al., 2016 [28]	Zirconia	After the extraction, the tooth was modified with light cured composite material to receive the crown afterward. Macro-retentions were designed on the root surface only on the mesial and distal surface with light cured flowable composite material. The modified root was laser scanned	Milling	Sandblasting
Pour et al., 2017 [29]	Titanium (root) Zirconia (abutment)	Impressions of the maxilla and a digital volume tomography (DVT) were taken. The DVT data with the impression used to determine the exact dimensions of the implant, were sent to Natural Dental Implants (NDI). Utilizing the 3D data derived from the DVT and the digitized casts, NDI designed and fabricated a patient-specific root-analogue immediate implant with a predesigned abutment. Additionally, microretentions were designed on the implant surface	Milling Materials bonded with a glass solder for sealing the interface between the implant and abutment	Radiation Acid etching Sandblasting (on the titanium part only)
Moin et al., 2018 [30]	Grade IV titanium (root) Zirconia (abutment)	After patient DICOM files acquisition and STL files of stone casts and bite registrations, a 3D envelope was created for the selected tooth representing the extension of the root, alveolar bone, marginal bone level, gingival margins, adjacent and antagonist dental structures, and anatomical structures. Within this 3D envelope, CAD designs of the root analogue implant were made consisting of root/implant portion and an abutment portion	Milling The two parts were bonded with a glass solder	Sandblasting Acid-etching (on the titanium part only)

**Table 3:** Summary of the clinical trials/case reports parameters and results.

Author/Year	Type of Study	Subjects *	Implant Per Patient	Tooth	Time between Extraction and Implantation	Implant Placement Details	Time between Placement and Reconstruction	Follow-Up	Technical and/or Biological Complications; Overall Performance
(Lundgren, et al., 1992) [11]	Pre-clinical trial (beagle dogs)	4 (2–3-years-old)	8	Variable	Dog 1–3 (2 weeks) Dog 4 (0 day)	Intra-alveolar soft tissue was removed, and the bone walls were not scraped. The implants were immediately placed. The mucoperiosteal flaps were repositioned and sutured	2 months	3 years	2 implants were lost in the first week. 30 implants were retained in the socket (of these, 2 were denuded because of late mucosal perforation but remained clinically stable). The remaining 28 implants fulfilled the clinical criteria for osseointegration
(Kohal et al., 1997) [17]	Pre-clinical trial (Macaca fascicularis)	3	4	Upper central and lateral incisors	0 day	The implants were tapped into their respective socket The mucoperiosteal flaps were repositioned and sutured	-	6 months	Buccal bone fracture at the time of implantation. Some of the implants could not be inserted to the intended depth. Four implant exposures (6 days, 8 days, 9 days and 2.5 months). None of the implants was surrounded by soft connective tissue. None of the implants were lost and all were clinically stable at the end of the experiment
(Heydecke et al., 1999) [18]	Case report (human)	1 (45, gender not specified)	1	Max. Sn. lateral incisor	1 day	The implant was placed into the socket using the attached insertion bar under finger pressure and subsequent tapping with a hammer and a mallet. Primary stability was checked with the handles of 2 dental mirrors. The insertion bar was removed and a custom-made healing cap was placed	6 months	No data	Bony resorption and buccal soft tissue recession The final result was compromised by metal shining through the thin gingiva
(Pirker and Kocher 2008) [19]		1 (63, gender not specified)	1	1st max. Dx premolar	4 days	The implant was placed into the socket under finger pressure and subsequent gentle tapping with a hammer and a mallet. Primary stability was achieved as checked by palpation and percussion	4 months	2 years	Stable implant. No changes on the peri-implant marginal bone level. No bleeding No signs of periodontitis nor bone resorption
(Pirker and Kocher 2009a) [20]	Clinical trial (human)	18 Group A (4 F, 2 M, 27–60-years-old) Group B (4 F, 8 M, 27–65-years-old)	1	Variable	Group A 1–4 days Group B 1–8 days		Group A No restoration Group B 3–13 months	2 years	Primary implant stability was achieved in all patients and no complications, such as swelling, inflammation, bleeding and pain (Group A) 5 implants were lost within 26–128 days (Group A). Implants were lost suddenly without prior pain or infection (Group A). Implant lost after 624 days. However, the lack of osseointegration was already observed on day 18 (Group B) All 11 remaining implants healed uneventfully with no complications (Group B). Soft tissue retraction ranged from 0–1.5 mm within the first year and remained stable thereafter. Many implants (58%) had no observed soft tissue retraction and maintained an aesthetic gingival architecture (Group B). There was no wound infection, no signs of periodontitis, and no implant mobility/dislocation (Group B)
(Pirker and Kocher 2009b) [21]	Case report	1 (27 M)	1	Dx. lateral maxillary incisor	7 days		3 months	15 months	Stable implant, unchanged peri-implant marginal bone level. No bleeding. Excellent aesthetic result. No signs of periodontitis nor bone or soft tissue recession
(Pirker et al., 2011) [22]	Case report	1 (50 F)	1	1st mand. Sn. molar			4 months	2.5 years	Stable implant, unchanged peri-implant marginal bone level. Complete apical peri-implant ossification with no signs of peri-implantitis
(Mangano et al., 2012) [23]	Case report	1 (55 M)	1	1st max. Dx. premolar	0 day		3 months	1 year	Primary stability was achieved, due to the perfect correspondence between the implant and the post-extraction socket The implant was still in function after a one-year follow-up The implant was stable, with no signs of infection, unchanged peri-implant marginal bone level and no peri-implant radiolucency The radiographic profile of the implant–crown complex was very similar to that of a natural tooth No prosthetic complications. The prosthetic restoration showed optimal functional and aesthetic integration
(Figliuzzi and Mangano 2012) [24]	Case report	1 (50 F)	1	2nd max. Dx premolar	0 day	The implant was placed into the socket under finger pressure and subsequent gentle tapping with a hammer and a mallet. Primary stability was achieved as checked by palpation and percussion	3 months	1 year	Implant in function after one year. The implant was stable with no signs of infection. Good conditions of the peri-implant tissues. Unchanged peri-implant marginal bone level and no peri-implant radiolucency. No prosthetic complications
(Mangano et al., 2014) [25]	Clinical trial	15 (8 M, 7 F, 39–55-years-old)	1	Premolars (8 max; 7 mand)	0 day				No implants were lost, leading to a survival rate of 100%. All implants were stable with no signs of infection. Unchanged peri-implant marginal bone level and no peri-implant radiolucency. The radiographic profile of the implant–crown complex was very similar to that of natural teeth. No prosthetic complications
(Pirker and Kocher 2015) [26]	Case report	1 (41 F)	1	Maxillary 2nd Sn. molar	6 day		7 months	3 years	The implant completely filled the extraction socket, ensuring perfect osseointegration. Unchanged peri-implant marginal bone levels. No signs of periodontitis, bone resorption nor bleeding. Excellent aesthetic result
(Figliuzzi et al., 2016) [27]	Case report	1 (45 M)	1	Lateral Dx. max. incisor	0 days	The implant was gently inserted in the socket using a little percussion hammer. Primary stability was achieved, as a consequence of the congruence between the implant and the socket. Then, sutures were positioned	3 months	1 year	After one year, the implant was still in function. No biological complications were reported. The peri-implant tissues were mature and stable. Little or no peri-implant bone loss, and no soft tissue recession
(Patankar et al., 2016) [28]	Case report	1 (22 F)	1	Dx. mand. 1st premolar	3 days	The implant was placed into the socket under finger pressure and subsequent gentle tapping with a hammer and a mallet. Primary stability was achieved as checked by palpation and percussion	4 months	18 months	Stable implant. Unchanged peri-implant marginal bone level and complete apical peri-implant ossification. No signs of peri-implantitis and no bleeding
(Pour et al., 2017) [29]	Case report	1 (35 F)	1	Max. Sn. central incisor	0 day	The implant was inserted and seated with cautious tapping into the socket and buccal augmentation was achieved with Bio-Oss for stabilizing the tissue architecture. The relief cut was sewn up with three single button sutures	6 months	16 months	Satisfactory aesthetics and stability of the surrounding tissues. Stability of the bone and implant functionality observed
(Moin et al., 2018) [30]	Clinical trial	5	1	Premolars	0 day	The implant was placed into the socket under finger pressure and subsequent gentle tapping with a hammer and a mallet. Primary stability was achieved as checked by palpation and percussion	3 months	1 year	In one patient, the implant showed mobility and symptoms of peri-implant infection after 4 weeks. The implant was removed at the 12 months evaluation, all remaining implants were successful. Two patients showed an absence of buccal bone around the implant. Healthy mucosal appearance in all remaining implants

* Remarks: 00A refers to age (years) and gender of patients (e.g., 45F is a 45 year old female).

## Data Availability

Not applicable.

## References

[1] Baldi D., Lombardi T., Colombo J., Cervino G., Perinetti G., Di Lenarda R., Stacchi C. (2018). Correlation between insertion torque and implant stability quotient in tapered implants with knife-edge thread design. Biomed. Res. Int. Hindawi.

[2] Malet J., Mora F., Bouchard F. (2012). Implant Dentistry at a Glance.

[3] Lindhe J., Karring T., Lang N.P. (2003). Clinical Periodontology and Implant Dentistry.

[4] Chen X., Xie L., Chen J., Du R., Deng F. (2012). Design and fabrication of custom-made dental implants. J. Mech. Sci. Technol..

[5] Giudice A., Bennardo F., Antonelli A., Barone S., Wagner F., Fortunato L., Traxler H. (2020). Influence of clinician’s skill on primary implant stability with conventional and piezoelectric preparation techniques: An ex-vivo study. J. Biol. Regul. Homeost. Agents..

[6] Chen J., Zhang Z., Chen X., Zhang C., Zhang G., Xu Z. (2014). Design and manufacture of customized dental implants by using reverse engineering and selective laser melting technology. J. Prosthet. Dent..

[7] Regish K.M., Sharma D., Prithviraj D.R. (2013). An overview of immediate root analogue zirconia implants. J. Oral. Implantol..

[8] Cicciù M., Cervino G., Milone D., Risitano G. (2018). FEM Investigation of the Stress Distribution over. Mandibular Bone Due to Screwed Overdenture Positioned on Dental Implants. Materials.

[9] Dantas T.A., Carneiro Neto J.P., Alves J.L., Vaz P.C.S., Silva F.S. (2020). In silico evaluation of the stress fields on the cortical bone surrounding dental implants: Comparing root-analogue and screwed implants. J. Mech. Behav. Biomed. Mater..

[10] Hodosh M., Shklar G., Povar M. (1974). The porous vitreous carbon/polymethacrylate tooth implant: Preliminary studies. J. Prosthet. Dent. Mosby.

[11] Lundgren D., Rylander H., Andersson M., Johansson C., Albrektsson T. (1992). Healing of root analogue titanium implants placed in extraction sockets. An experimental study in the beagle dog. Clin. Oral Implant. Res..

[12] Pessanha-Andrade M., Sordi M.B., Henriques B., Silva F.S., Teughels W., Souza J.C.M. (2018). Custom-made root-analogue zirconia implants: A scoping review on mechanical and biological benefits. J. Biomed. Mater. Res. Part B Appl. Biomater..

[13] Osman R.B., Swain M.V. (2015). A critical review of dental implant materials with an emphasis on titanium versus zirconia. Materials.

[14] Zühlke A., Gasik M., Shahramian K., Närhi T., Bilotsky Y., Kangasniemi I. (2021). Enhancement of gingival tissue adherence of zirconia implant posts: In vitro study. Materials.

[15] Matinlinna J.P. (2014). Handbook of Oral Biomaterials.

[16] Moher D., Liberati A., Tetzlaff J., Altman D. (2009). Preferred Reporting Items for Systematic Reviews and Meta-Analyses: The PRISMA Statement. PLoS Med. Public Libr. Sci..

[17] Kohal R., Hürzeler M., Mota L., Klaus G., Caffesse R., Strub J. (1997). Custom-made root analogue titanium implants placed into extraction sockets: An experimental study in monkeys. Clin. Oral Implant. Res..

[18] Heydecke G., Kohal R., Gläser R. (1999). Optimal esthetics in single-tooth replacement with the Re-Implant system: A case report. Int. J. Prosthodont..

[19] Pirker W., Kocher A. (2008). Immediate, non-submerged, root-analogue zirconia implant in single tooth replacement. Int. J. Oral Maxillofac. Surg..

[20] Pirker W., Kocher A. (2009). Immediate, non-submerged, root-analogue zirconia implants placed into single-rooted extraction sockets: 2-year follow-up of a clinical study. Int. J. Oral Maxillofac. Surg..

[21] Pirker W., Kocher A. (2009). True Anatomic Immediate Dental Implant Method. Implants.

[22] Pirker W., Wiedemann D., Lidauer A., Kocher A. (2011). Immediate, single stage, truly anatomic zirconia implant in lower molar replacement: A case report with 2.5 years follow-up. Int. J. Oral Maxillofac. Surg..

[23] Mangano F., Cirotti B., Sammons R., Mangano C. (2012). Custom-made, root-analogue direct laser metal forming implant: A case report. Lasers Med. Sci..

[24] Figliuzzi M., Mangano F. (2012). A novel root analogue dental implant using CT scan and CAD/CAM: Selective laser melting technology. Int. J. Oral Maxillofac. Surg..

[25] Mangano F., De Franco M., Caprioglio A., MacChi A., Piattelli A., Mangano C. (2014). Immediate, non-submerged, root-analogue direct laser metal sintering (DLMS) implants: A 1-year prospective study on 15 patients. Lasers Med. Sci..

[26] Pirker W., Kocher A. (2015). Root analog zirconia implants: True anatomical design for molar replacement—A case report. Int. J. Periodontics Restor. Dent..

[27] Figliuzzi M., Giudice A., Rengo C., Fortunato L. (2016). A direct metal laser sintering (DMLS) root analogue implant placed in the anterior maxilla. Case report. Ann. Ital. Chir..

[28] Patankar A., Kshirsagar R., Patankar S., Pawar S. (2016). Immediate, Non Submerged Root Analog Zirconia Implant in Single Rooted Tooth Replacement: Case Report with 2 years Follow Up. J. Maxillofac. Oral. Surg..

[29] Pour R., Randelzhofer P., Edelhoff D., Prandtner O., Rafael C., Liebermann A. (2017). Innovative Single-Tooth Replacement with an Individual Root-Analog Hybrid Implant in the Esthetic Zone: Case Report. Int. J. Oral Maxillofac. Implant..

[30] Moin D., Hassan B., Wismeijer D. (2018). Immediate Nonsubmerged Custom Root Analog Implants: A Prospective Pilot Clinical Study. Int. J. Oral Maxillofac. Implant..

[31] Andersson M., Bergman B., Bessing C., Ericson G., Lundquist P., Nilson H. (1989). Clinical results with titanium crowns fabricated with machine duplication and spark erosion. Acta Odontol. Scand..

[32] Strub J.R., Kohal R.J., Klaus G., Ferraresso F. (1997). The Re implant^®^ system for immediate implant placement. J. Esthet. Restor. Dent..

[33] Rimondini L., Gasik M., Vrana E.N. (2018). Bacterial Attachment and Biofilm Formation on Biomaterials: The case of dental and orthopaedic implants. Biomater Immune Response Complicat Mech Immunomodulation.

[34] Gasik M., Mellaert L., van Pierron D., Braem A., Hofmans D., Waelheyns E.D., Vleugels J. (2012). Reduction of biofilm infection risks and promotion of osteointegration for optimized surfaces of titanium implants. Adv. Health Mater..

[35] Shahramian K., Gasik M., Kangasniemi I., Walboomers X.F., Willberg J., Abdulmajeed A., Närhi T. (2020). Zirconia implants with improved attachment to the gingival tissue. J. Periodontol..

[36] Niinomi M. (2008). Biologically and Mechanically Biocompatible Titanium Alloys. Mater. Trans. Japan Inst. Met. Mater..

[37] Reveron H., Fornabaio M., Palmero P., Fürderer T., Adolfsson E., Lughi V., Chevalier J. (2017). Towards long lasting zirconia-based composites for dental implants: Transformation induced plasticity and its consequence on ceramic reliability. Acta Biomater..

[38] Bona ADella Pecho O.E., Alessandretti R. (2015). Zirconia as a dental biomaterial. Materials.

[39] Quirynen M., Abarca M., Van Assche N., Nevins M., van Steenberghe D. (2007). Impact of supportive periodontal therapy and implant surface roughness on implant outcome in patients with a history of periodontitis. J. Clin. Periodontol..

[40] Krishna Alla R., Ginjupalli K., Upadhya N., Shammas M., Krishna Ravi R., Sekhar R. (2011). Surface Roughness of Implants: A Review. Trends Biomater. Artif. Organs.

[41] Saghiri M.A., Asatourian A., Garcia-Godoy F., Sheibani N. (2016). The role of angiogenesis in implant dentistry part I: Review of titanium alloys, surface characteristics and treatments. Med. Oral Patol. Oral.

[42] Depprich R., Zipprich H., Ommerborn M., Naujoks C., Wiesmann H.-P., Kiattavorncharoen S., Handschel J. (2008). Osseointegration of zirconia implants compared with titanium: An in vivo study. Head Face Med..

[43] Sennerby L., Dasmah A., Larsson B., Iverhed M. (2005). Bone tissue responses to surface-modified zirconia implants: A histomorphometric and removal torque study in the rabbit. Clin. Implant Dent. Relat. Res..

[44] Roccuzzo M., Bonino L., Dalmasso P., Aglietta M. (2014). Long-term results of a three arms prospective cohort study on implants in periodontally compromised patients: 10-year data around sandblasted and acid-etched (SLA) surface. Clin. Oral Implant. Res..

[45] Greenstein G., Cavallaro J., Romanos G., Tarnow D. (2008). Clinical Recommendations for Avoiding and Managing Surgical Complications Associated With Implant Dentistry: A Review. J. Periodontol..

[46] Rompen E., Domken O., Degidi M., Pontes A.E.P., Piattelli A. (2006). The effect of material characteristics, of surface topography and of implant components and connections on soft tissue integration: A literature review. Clin. Oral Implant. Res. Clin. Oral Implant. Res..

[47] Davidowitz G., Kotick P.G. (2011). The Use of CAD/CAM in Dentistry. Dent. Clin..

[48] Susic I., Travar M., Susic M. (2017). The application of CAD / CAM technology in Dentistry. IOP Conf. Ser. Mater. Sci. Eng..

[49] Goto T. (2014). Osseointegration and dental implants. Clin. Calcium..

[50] Dantas T.A., Abreu C.S., Costa M.M., Miranda G., Silva F.S., Dourado N., Gomes J.R. (2017). Bioactive materials driven primary stability on titanium biocomposites. Mater. Sci. Eng. C..

[51] Moin D.A., Hassan B., Wismeijer D. (2016). A Patient Specific Biomechanical Analysis of Custom Root Analogue Implant Designs on Alveolar Bone Stress: A Finite Element Study. Int. J. Dent..

[52] Lin D., Li Q., Li W., Swain M. (2009). Dental implant induced bone remodeling and associated algorithms. J. Mech. Behav. Biomed. Mater. J. Mech. Behav. Biomed. Mater..

[53] Katz J.L., Misra A., Marangos O., Ye Q.S.P., Peterson D.R., Bronzino J.D. (2015). Mechanics of hard tissue. Biomechanics: Principles & Practices.

[54] Chen J., Ahmad R., Li W., Swain M., Li Q. (2015). Biomechanics of oral mucosa. J. R. Soc. Interface..

[55] Marques A., Miranda G., Faria D., Pinto P., Silva F., Carvalho Ó. (2019). Novel design of low modulus high strength zirconia scaffolds for biomedical applications. J. Mech. Behav. Biomed. Mater..

[56] Dantas T.A., Pinto P., Vaz P.C.S., Silva F.S. (2020). Design and optimization of zirconia functional surfaces for dental implants applications. Ceram Int..

[57] Raikar S., Talukdar P., Kumari S., Panda S.K., Oommen V.M., Prasad A. (2017). Factors affecting the survival rate of dental implants: A retrospective study. J. Int. Soc. Prev. Community Dent..

